# Exploring Low-Income Families' Financial Barriers to Food Allergy Management and Treatment

**DOI:** 10.1155/2014/160363

**Published:** 2014-02-17

**Authors:** Leia M. Minaker, Susan J. Elliott, Ann Clarke

**Affiliations:** ^1^School of Public Health and Health Systems, University of Waterloo LHN 1711, 200 University Avenue West, Waterloo, ON, Canada N2L 3G1; ^2^Faculty of Applied Health Sciences, University of Waterloo BMH 3115, 200 University Avenue West, Waterloo, ON, Canada N2L 3G1; ^3^Department of Medicine, McGill University, Room 101, Lady Meredith House, 1110 Pine Avenue West, Montreal, QC, Canada H3A 1A3

## Abstract

*Objectives*. Low-income families may face financial barriers to management and treatment of chronic illnesses. No studies have explored how low-income individuals and families with anaphylactic food allergies cope with financial barriers to anaphylaxis management and/or treatment. This study explores qualitatively assessed direct, indirect, and intangible costs of anaphylaxis management and treatment faced by low-income families. *Methods*. In-depth, semistructured interviews with 23 participants were conducted to gain insight into income-related barriers to managing and treating anaphylactic food allergies. *Results*. Perceived direct costs included the cost of allergen-free foods and allergy medication and costs incurred as a result of misinformation about social support programs. Perceived indirect costs included those associated with lack of continuity of health care. Perceived intangible costs included the stress related to the difficulty of obtaining allergen-free foods at the food bank and feeling unsafe at discount grocery stores. These perceived costs represented barriers that were perceived as especially salient for the working poor, immigrants, youth living in poverty, and food bank users. *Discussion*. Low-income families report significant financial barriers to food allergy management and anaphylaxis preparedness. Clinicians, advocacy groups, and EAI manufacturers all have a role to play in ensuring equitable access to medication for low-income individuals with allergies.

## 1. Introduction

Food allergies affect 4–8% of people in the Western world [1–3]. Anaphylactic food allergies are rapid in onset and can be fatal if treatment is delayed [4]; thus, complete dietary avoidance of the allergen and timely access to treatment are essential. Three studies found that children from low-income families face barriers to appropriate anaphylaxis management [5–7]. Conversely, one study found that associations between allergy severity and health care utilization did not differ by poverty status [8] and another study found no conclusive associations between the epinephrine autoinjectors (EAIs) availability and sociodemographic factors [9].

A recent study on the economic impact of childhood food allergy in the United States found the overall economic cost of food allergy to be almost $25 billion annually [10]. This study found that costs borne by families affected by food allergies include lost labour productivity, out-of-pocket costs, and opportunity costs [10]. Costs associated with food allergies are described as direct, indirect, or intangible [11–13]. Direct costs are financial costs incurred as a result of the food allergy (including inpatient admissions, outpatient visits, medication costs, and the cost of suitable foods) [14]. Indirect costs reflect time spent in various activities as a result of food allergy, including lost productivity and opportunity costs. Intangible costs are losses of utility, such as experiences of pain, suffering, and grief, and can be measured by self-reported quality of life and well-being [12, 15]. One recent study on the intangible costs of food allergy in four European countries found that patients with food hypersensitivity reported lower well-being than the control group. The authors note, however, that well-being was positively related to income and, thus, income compensation might improve welfare among food hypersensitive individuals [15]. Low-income individuals are differentially affected by the costs of food allergy, since “… people with allergy who also have low incomes may have greater difficulties in access to [allergen-free] food.” [11, page 999]. It is intuitive that absolute costs of allergy medication or allergen-free foods pose a disproportionate burden to low-income families. Although these costs are conventionally assessed quantitatively, exploring qualitatively assessed direct, indirect, and intangible costs of food allergies among low-income families is important for three reasons. First, low-income individuals may have poorer access to anaphylaxis treatment [5, 6] and may therefore be at high risk of allergy-related harms. Second, understanding barriers to anaphylaxis management or treatment faced by low-income individuals can facilitate the development of valid quantitative surveys intended to describe the overall scope of food allergies and treatment accessibility. Third, exploring how low-income families cope with or manage anaphylactic allergies has both clinical and policy implications.

Qualitative research has “enormous potential to make a distinctive contribution to knowledge about allergy management and to provide insights that quantitative studies cannot.” [16, page 1117]. Although extant research assesses direct, indirect, and intangible costs quantitatively, this study explores these costs qualitatively from the perspective of low-income families, which is a current gap in the allergy literature. Findings from in-depth interviews with key informants and low-income families affected by food allergy begin to fill the gaps in our understanding around coping and management for this vulnerable population. The purpose of this study was to take an in-depth look at the direct, indirect and intangible costs that are particularly burdensome for low-income families in terms of anaphylactic food allergy management and treatment.

## 2. Methods

In-depth, semi-structured interviews with key informants (*n* = 10) and low-income individuals affected by food allergy (*n* = 13) were conducted in southwestern Ontario, Canada. In Ontario, the provincial health insurance plan covers the cost of physician and other “medically necessary” services. Of note, drugs (including EAIs) are not covered by the provincial health insurance plan. However, individuals who are receiving social assistance or are 65 years of age or older are eligible for drug coverage through the provincial government. Individuals who are not receiving social assistance either have private drug insurance through their employer or pay out of pocket for drugs. Key informants were recruited from agencies working with low-income families in a context relevant to food allergy (i.e., food procurement or medication access) (Table 1). Low-income participants were recruited via posters placed in these targeted agencies. Over half (54%) of participants were parents of allergic children; the remainder were allergic adults. Most (77%) participants were on social assistance, which included drug benefits (Table 2). All allergic individuals had been prescribed EAIs for their food allergies. Verification of diagnosis was not requested from participants' doctors because many participants reported not having a family doctor. All participants self-identified as being low income and were using community services offered specifically to low-income people. The first author conducted all interviews, which continued until saturation was reached. Interviews were transcribed verbatim for subsequent thematic analysis using NVivo 10 [17]. Interrater reliability assessment between two transcripts achieved reliability of 0.70 [18]. After a theme code set was developed, two researchers conducted intercoder tests to determine levels of coding agreement (70% agreement or higher is considered acceptable) to ensure confidence in the coding scheme [18]. Ethical clearance was received from the University of Waterloo. 

## 3. Results

### 3.1. Perceived Direct Costs

#### 3.1.1. Medication

Over half (54%) of low-income participants and 20% of key informants noted the high cost of EAIs as a barrier to anaphylaxis treatment for low-income families. EAIs cost about USD$100, which many allergists consider to be high in relation to minimum wage and average daily earnings [19]. Participants often kept expired EAIs rather than replacing them promptly; cost also affected their decision whether to purchase at all:

*When you have an anaphylactic reaction you don't just have the fear in that whole process and then your body feeling terrible for two to five days after, you have also just lost $100 or $200. (Allergic female)*

One mother, unsure of her son's EAI expiry date, spoke of the impact her financial situation had on replacing expired EAIs:

*So coming up with a hundred dollars to replace it again, that is probably one of the reasons why I haven't checked to see if it is expired. (Mother of allergic toddler)*

Keeping expired EAIs was reported by 31% of low-income participants. One woman described her experience of living as a homeless youth with an anaphylactic allergy thus,

*They expire. Yeah I always carry around all of mine, and the first ones I ever try would be the expired ones, just to try… it will save… like money, while you are saving your life, right. Sounds terrible to say it that way but it is really true. Just with the cost of an EpiPen, like that is a lot of money especially if you are like fifteen and living on the street. So it is like take the expired ones first and then usually they work and if they don't take the other one. (Allergic female)*

Some participants reported retaining an inadequate number of EAIs or not having one at all due to cost. When asked how many EAIs she had for her tree-nut-allergic son, a low-income mother said, “Just the one. I can only afford one.”

These experiences were echoed by the perceptions of an allergist:

*I think there is a considerable amount… especially in the lower income, that will make a decision based on, “if I use that now, I am not going to have it for the next ten months.” And a $100 item may be a big deal for somebody who is generating… income below the poverty line. So I think that their reliance would still be on an antihistamine and prolonged care at home. (Allergist)*

This is problematic because the first line of anaphylaxis treatment is epinephrine; antihistamines are not a recommended treatment [20], although they may be perceived as a low-cost alternative. Several participants confirmed the allergist's perception. One woman said:

*… my first initial thing is to take Benadryl. I always take Benadryl. I use liquid, so that way it gets into your system faster, as opposed to pill form. (Allergic female)*

A peanut-allergic woman who had been hospitalized numerous times for anaphylactic reactions and who considered EAIs to be a “waste of money” considered antihistamines a “life line” for treating her peanut allergy.

#### 3.1.2. Procuring Safe Foods

Less than half (43%) of respondents perceived no price differential for allergen-free foods, while 35% did:

*I think they are more expensive… because the factories have to make sure that you know there is no allergens in it… because we had to figure out how to make this without eggs, we are going to charge you two bucks more. (Allergic female)*

Five participants (22%) thought that cost was dependent on the type of allergen-free food:

*It depends what you are looking for—wheat stuff—like wheat free stuff is expensive. (Mother of an allergic child)*

Beyond the direct cost of specialty food items, participants differed in their opinions of how their overall grocery bill changed subsequent to an allergy diagnosis. Participants who reported that their grocery bill remained unchanged generally referred to the allergen being a previously infrequent purchase with no overall impact on their grocery bill. Overall, no clear consensus on costs associated with procuring allergen-free foods emerged among participants.

About a quarter (23%) of low-income participants reported that their weekly grocery bill decreased since the allergy diagnosis due to a reduction in purchasing more expensive processed (and potentially cross contaminated) foods or due to the elimination of purchasing expensive, allergenic foods (e.g., cashews). Regarding prediagnosis grocery bills, one woman explained,

*Probably more, just because there were more things that I could buy. And then I had to start like making my own I guess… you know, which could be cheaper. (Mother of an allergic child)*

Finally, two participants (15%) described increased grocery costs after diagnosis:

*I would say our grocery budget… I would have to estimate… 15% increase overall simply because we buy brand name. We can't buy bulk… being a low-income family that is where it affects us… (Father of an allergic child)*

#### 3.1.3. Direct Costs Resulting from Misinformation

A significant proportion (46%) of low-income participants reported misinformation related to social support programs and medical insurance coverage. Some misinformation resulted in direct costs to participants:

*Ontario Works [Ontario's Welfare System] won't pay for an epi-pen. It isn't covered through their benefit program. (Allergic female)*

This statement was in direct contradiction to information provided by a key informant employed by Ontario Works who noted how common misinformation was:

*We can't tell each person everything that they can qualify for. You say a drug card and you hope they kind of put it all together. So with allergies and everything you hope people will come to you and say, “I have this problem.” And then we can talk about just that one specific one, but it is hard to get enough of the information out there… (Social assistance key informant)*

One participant who knew she required an EAI but was unaware that it would be covered by her social assistance drug plan rationalized her lack of EAI by exaggerating the direct costs,

*And I haven't gotten one now, because they are like a hundred and fifty bucks a piece… Every six months you have to renew it.*

Other participants who were unaware that they qualified for EIA coverage through Ontario Works spoke of “taking care of themselves” so they would not require an EIA:

*And so it is sometimes like wasting money… but I think as an adult now I will take much better care of myself and I just don't eat nothing [if] I don't know who cooked it. (Allergic female)*

In the event of an unintentional exposure to an allergen, two participants who did not have EAIs due to the perceived high cost reported using emergency services instead of an EAI.

### 3.2. Perceived Indirect Costs 

#### 3.2.1. Lack of a Consistent Family Doctor

There were few indirect costs associated with anaphylactic food allergies that were unique to low-income families. This is because indirect costs, including lost labour productivity, obtaining health care, and time spent shopping for safe foods, are actually higher for people who earn more money [10, 12]. Only one indirect cost emerged as unique to low-income individuals with anaphylactic food allergies: 17% of participants referred to low-income groups having poorer continuity of care by family doctors, which may result in increased opportunity costs in terms of being unnecessarily re-tested for allergies.

*… for folks who are dependent on urgent care, which is a lot of people, they just don't have family doctors… that would be… terrible to try and navigate. (Employment centre key informant)*

One woman said,

*You have to have some sort of identification proving your allergies. We have a new family doctor, and I have never had a scratch test done with him, so he does not have anything really on record about my allergies… (Allergic female)*

### 3.3. Perceived Intangible Costs

#### 3.3.1. Stress

Almost 40% of participants (5 key informants and 4 low-income respondents) reflected on the challenge of eating an adequate, healthy diet due to a lack of money. Many participants who relied on food banks felt stress due to the additional difficulty of obtaining allergen-free foods from the food bank (48% of participants):

*Well a grocery store, you can pick and choose what you are getting. When it comes to the food hamper, you have got to take what you get, right… Even though you put down specifically in a yellow highlight pencil, or whatever, that [I'm] severely allergic to nuts, I still get it. (Allergic male)*

One woman further explained how reporting allergies can actually decrease the overall amount of food that can be obtained from a food bank,

*… they don't give you anything extra. If you are allergic to something, you just don't get it. They don't substitute it with anything, because they can only give you what they have, right… we have never said okay we are allergic to this, and they will give us something else. No, it is just you just don't get it. You get whatever else is left in your hamper. (Allergic female)*

#### 3.3.2. Feeling Unsafe

Almost one-third of participants reported feeling less safe at discount supermarkets relative to regular supermarkets because of perceived poor food availability and cross-contamination that participants thought was worse in discount supermarkets. In terms of product availability:

*I know they are going to have a wide variety of brands to choose from, so it makes it easier for me. Whereas [discount supermarket] might get stuff from the States, so it takes much longer to go through all the labels at [discount supermarket] than it would at [regular supermarket]. (Mother of allergic child)*

Respondents also pointed to employees practices:

*Because I don't feel that [discount supermarket] probably deals with their produce as well as [regular supermarket] does. Like at [regular supermarket] you can see that how often the employees wash their hands. At [discount supermarket] they just touch this and they go touch that. I have watched them… they will pay their employees less. You know they are less worried about what kind of produce they have, and what they're touching and what they are doing with it. (Allergic female)*

These data reveal trade-offs between direct and intangible risks: participants who shopped at regular grocery stores recognized that spending more on groceries (increased direct costs) reduced their fear of cross-contamination (decreased intangible costs).

### 3.4. Especially “at Risk” Groups

Four distinct low-income groups were described by participants as facing substantial barriers to proper anaphylaxis management and treatment. Of note, groups identified by participants were not groups with which the participants self-identified (e.g., only Canadian-born participants identified that newcomers to Canada may face additional barriers to food allergy management). These were the working poor with no or inadequate health insurance (22% of participants), newcomers to Canada (i.e., foreign immigrants) (17%), food bank users (13%), and youth living in poverty (9%). Both allergists and several low-income participants identified the working poor as being likely unable to afford EAIs. One allergist said,

*If you are a working poor person that is making minimum wage or a little bit more, they may not have the drug benefits that the government offers and their jobs might not be “good enough” to have a drug plan. They would fall through the cracks. So I suspect that the majority of these people will not buy themselves EpiPens because they are just too expensive.*

Newcomers to Canada were another low-income group considered to be especially at risk:

*New Canadians would be on very limited incomes, but they might have like food sensitivities and like, “My child is very, very sick and has to go to the hospital”, but anaphylactic just isn't a familiar word and allergy even is not like a familiar word. (Employment centre key informant)*

A newcomer who participated in this research had difficulty articulating her son's allergies:

*To eggs, some of the nuts… cashews, and there is another one. I don't know what the name of that. It is, it looks similar like walnut, but it is not walnut. It is round and kind of this much big. (Mother of allergic child)*

This mother knew no one in her home country with a food allergy and had an EAI trainer device but was unsure what it was or how to use it. She did not have an EAI for her son.

Participants' stress related to the difficulty of obtaining allergen-free foods at the food bank (discussed above) explains why several participants noted that food bank users qualify as another “at risk” group. In addition to the perceived potential for cross-contamination at the food bank, one woman said,

*It frustrates me that the food bank never says nut-free products when they are looking for food. They always advertise, “We need baby cereal. We need peanut butter. We need this.” They never say peanut-free anything. (Mother of an allergic child)*

Finally, two key informants related their concerns about youth with anaphylactic allergies who lived in poverty. One key informant was concerned about youth living in poverty being embarrassed to vocalize their allergy-related concerns,

*I think it is embarrassing for a lot of the kids. I don't want to ask for food from you, because I don't want you to know I am hungry, like that I have no money, and that my parents have kicked me out. A lot of them don't know a nice way of saying, just tell me what is in this, you know, so they don't. (Social assistance key informant)*

Another key informant shared knowledge of how a street youth with anaphylactic food allergies might cope:

*I think too, people on the street share EpiPens or sell expired ones or like there is kind of a small black market. [EpiPens are] probably $20–40. $20 probably for an expired one. $40–60 maybe for one that is not expired. Something like that. A lot less, but pretty much 50% less. (Employment centre key informant)*

Youth in extreme poverty who have no access to drug insurance may have to rely on drastic measures to access medication—in this case, medication that is much more affordable once it has expired.

## 4. Discussion

The economic impact of food allergies is significant [10, 14, 15]. Out-of-pocket costs differentially impact low-income families. No studies to date have provided an in-depth exploration of how low-income families experience the direct, indirect, or intangible costs associated with food allergy management or treatment. Complementing the economic analyses that have described costs of food allergy, our qualitative study has described the *meaning* of the costs of food allergy (including how they cope with and manage the high cost of EAIs) among a group of low-income families. The qualitative approach we adopted highlighted discrepancies between low-income families' perceptions about barriers to managing food allergies and the actual availability of support (e.g., while Ontario's social assistance drug plan covers the cost of EAIs, many families perceived that the social assistance drug plan did not cover EAIs).

Low-income families and key informants perceive significant financial barriers to appropriate management and treatment of anaphylactic food allergies. These barriers can be broadly conceived as access to drugs and procuring safe foods. Our findings that low-income individuals are perceived to have low access to family doctors are contradicted by empirical evidence suggesting that, in countries where income-related inequities exist in the distribution of family doctor visits, it is often a propoor distribution [21]. That said, prorich income-related inequalities in access to specialists (such as allergists) are common [21, 22]. In terms of access to medication, in many jurisdictions, pharmaceuticals are not publicly insured. Therefore, working poor families (which comprise 25% of low-income Canadians under 65 years [23]) may be particularly at risk of having inadequate access to medication. Almost 70% of our low-income sample reported having an up-to-date EAI; in another study, 87% of a fairly well-educated sample had an up-to-date EAI [9] and 75% of children of wealthier families registered in a Peanut Allergy Registry reported having up-to-date EAIs (A. Clarke, unpublished observation). Although retaining expired EAIs is not practiced exclusively by low-income families, financial barriers to medication access should be addressed alongside educational campaigns for the broader allergic population. Indeed, the fact that some families would chance an anaphylactic reaction for themselves or their children due to the cost of an EAI is quite concerning.

Limitations of this study include the fact that all participants were from Ontario: experiences with allergy management and access to medication and allergen-free foods may differ in other jurisdictions. Although there were only 23 participants, the goal of qualitative research is to explore individuals' experiences rather than to generalize or verify hypotheses about causal relationships, and thus the small sample was not necessarily a limitation of the current study [24]. Strengths of this study are the in-depth interviews employed to gain insight into the experiences of low-income families affected by food allergies as well as the inclusion of both affected individuals and a number of key informants who represented a variety of stakeholder perspectives.

Perceived direct and intangible costs represented barriers to procuring safe foods for participants. Media reports suggest that food allergies greatly increase a family's food budget [25]; empirical evidence from the UK indicates that nut-allergic individuals pay an average of 11% more for a grocery basket than non-nut-allergic individuals [26]. These findings may explain increased grocery bills observed in Finland for families with allergic infants [13]. Moreover, the availability of suitable food options in budget supermarkets may be limited [27]. Finally, food bank use is increasing in Canada [28], the United States [29], and the UK [30]; these trends have implications for the safety of low-income individuals with anaphylactic food allergies.

The lack of robust findings around indirect costs of food allergy unique to low-income families reflects the fact that indirect costs like labour productivity are higher for people who make more money per hour relative to people who make less money per hour.

“At risk” groups, including the working poor, newcomers, food bank users, and youth in poverty, are especially important groups to target in terms of education and medication assistance. In terms of newcomers, however, it is likely that competency in the host country's language would moderate experiences of difficulty managing anaphylactic food allergies. Although many clinicians do not collect income data from participants, encouraging patients on social assistance to confirm their drug benefits to procure EAIs at low or no cost may be an important practice implication from this research. Clinicians, advocacy groups, policy makers, and EAI manufacturers all have a role to play in ensuring more equitable access to required medication for low-income individuals with anaphylactic food allergies. In addition, from a prevention perspective, securing allergen-free foods is equivalent to a “treatment.” Thus, providing additional funds to families to ensure their ability to secure allergen-free foods for their families may be another potential policy option.

## Figures and Tables

**Table 1:** Key informant sectors and job descriptions.

Number	Sector	Job description
1	Health care	Allergist
2	Health care	Allergist
3	Public health	Public health dietitian (schools)
4	Social services	Dietitian (low-income family programs)
5	Social services	Food bank
6	Social services	Food bank
7	Social services	Food bank
8	Social services	Employment support (working centre)
9	Social services	Ontario Works
10	Social services	Ontario Works

**Table 2:** Demographic data of low-income individuals or families.

Number	Child/adult	Male/female respondent	Male/female with allergy	On social support
1	Child	Female	Male	No
2	Child	Female	Female	Yes
3	Child	Female	Male	Yes
4	Child	Female	Male	Yes
5	Child	Female	Female	Yes
6	Child	Female	Female	Yes
7	Child	Male	Male	No
8	Adult	Female	Female	No
9	Adult	Female	Female	Yes
10	Adult	Female	Female	Yes
11	Adult	Female	Female	Yes
12	Adult	Female	Female	Yes
13	Adult	Male	Male	Yes

Totals	54% children	85% female	62% female	23% no

## Abbreviations

EAI: Epinephrine auto injector.
